# Assessment of ovarian tissues autografted to various body sites followed by IVM in mouse

**Published:** 2014-03

**Authors:** Mohammad Ali Khalili, Maryam Dehghan, Saeedeh Nazari, Azam Agha-Rahimi

**Affiliations:** 1 *Department of Biology and Anatomical Sciences, Research and Clinical Center for Infertility, Shahid Sadoughi University of Medical Sciences, Yazd, Iran.*; 2 *Neurobiomedical Research Center, Shahid Sadoughi University of Medical Sciences, Yazd, Iran.*; 3 *Research and Clinical Center for Infertility, Shahid Sadoughi University of Medical Sciences, Yazd, Iran.*

**Keywords:** *Ovarian transplantation*, *In vitro maturation*, *Mouse*

## Abstract

**Background:** Ovarian tissue transplantation is emerging technologies for fertility preservation. In addition, in vitro maturation (IVM) of oocytes retrieved from ovarian tissues may overcome the fertility defects in certain cases.

**Objective:** The aim was to evaluate the best site for ovarian tissue transplantation in mice. Also, feasibility of IVM of oocytes retrieved from auto grafted ovarian tissues was freshly assessed.

**Materials and Methods: **Hemi-ovaries from 6 weeks old mice were auto grafted into kidney capsule (K) versus the back muscle (B) and leg muscle (L) in a mouse auto graft model which was stimulated with gonadotrophins. Then ovarian grafts were recovered and processed histologically for follicle assessment compared with control, also the ability of oocytes to mature with IVM was studied 14 days after transplantation.

**Results: **Total follicle count was significantly higher in K-graft (3.5±3.17) and the antral follicles were only observed in K-site model. The number of retrieved immature oocytes as well as successful IVM in K-grafts was significantly higher than other groups (p=0.008, p=0.016).

**Conclusion:** The kidney capsule is a promising site for ovarian tissue auto graft in mice. This resulted in better follicular survival and IVM outcomes.

## Introduction

Ovarian tissue transplantation and in vitro maturation (IVM) of oocytes are two emerging technologies for fertility preservation of certain patients. Primary ovarian insufficiency is one of the side effects of cancer therapy in young women (1). It has been established that normal fertility can be restored by orthotopic grafting of fresh or frozen ovarian tissue (2). Revascularization of the ovarian grafts is generally influenced by several factors, including nerve growth factor (3). Liu *et al* showed normal ovarian functioning after transplantation of fresh or frozen-thawed ovaries from newborn mice to ovariectomized recipient mice (4). 

Previous study showed that the back muscle is a promising site for ovarian allograft in animal models, as birth of healthy offspring were reported after Intra-cytoplasmic sperm injection (ICSI) (5). Ovarian grafting to various body sites provides a model to study canine ovarian function (6). Several studies have shown that transplantation of ovarian tissues to various heterotropic locations could restore normal ovulatory functioning (7, 8).

Von Schonfeldt *et al* were the first to study the ovarian tissue transplantation in primates. Their findings demonstrated graft sustainment and development of follicles from prepubertal ovarian tissue (9). Ovarian grafts under the fascia of quadriceps femoris muscle facilitate access to the graft for oocyte collection (10). It is also found that primordial follicles were able to recruit preantral follicles during the period of transplantation and resulted in birth of live offspring (11). The study of heterotopic transplantation into the kidney capsules of ovariectomized mice also demonstrated that primordial follicles were capable of sustaining their developmental potential (12). 

Normal function of fresh ovarian grafts, contrary to initial expectation, indicated minimal oocyte loss from ischemic time (13). In their recent work, Soleimanian *et al* demonstrated that xenografting of cryoperserved human ovarian tissue into back muscle of mice obtained largest antral follicles containing mature oocytes (14). The previous study showed that oocytes undergoing IVM could successfully be used for intra follicular oocyte transfer (15). 

In assisted reproductive techniques, after ovarian stimulation approximately 15% of retrieved oocytes are immature. These oocytes are capable of maturing in vitro to be used for conventional IVF treatment in certain cases (16, 17). In young cancer survivors, ovarian grafts and IVM of primordial follicles may avoid the risk of cancer retransmission (18). Therefore, we autografted mouse hemi-ovaries into kidney capsule (K) versus the back muscle (B) and compared this sites to leg muscle site (L). In addition the grafted ovarian tissues were studied with both functional and histological techniques. Also, we studied graft functionality by IVM for retrieved immature oocytes. 

## Materials and methods

**Animals**

This was an experimental study, thirty female mice (25-30 g, 6 weeks old) purchased from* Research and Clinical Center for Infertility in Yazd.* The animals were randomly assigned into 2 groups of control and experimental. Animals were housed with 12/12h light-cycle with free access to water and food. The temperature was around 25^o^C with humidity of 45-55%. The study was approved by the Research and Clinical Center for Infertility, shahid sadoughi YAZD University of medical sciences Animal ethics committee. 

**Transplantation procedure**

**Figure 1:**
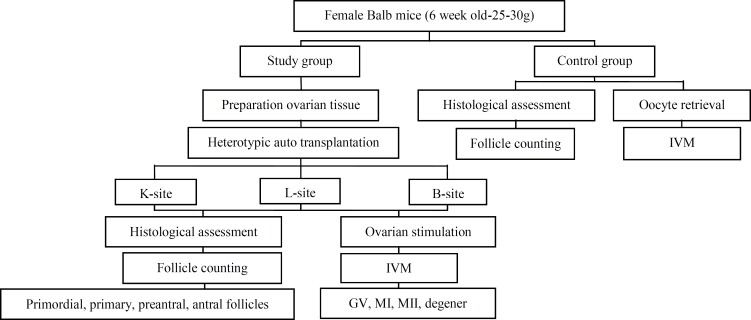
Flow chart to explain the study outline

Animals were anesthetized with ketamine (5mg/100g body weight; i.p.). Negative response to toe and tail pinch was used as an indication for anesthesia. During grafting, both ovaries were removed by cauterization at the top of the uterine horns. Then, the right ovary was cut approximately to half with surgical blade. The ovarian tissues (OTs) were collected in a 35 mm Falcon culture dish containing 2ml of pre-warmed MCDB 105+M199 medium (Sigma) with ratio of 1:1. Hemi-ovaries from the right ovary were transplanted at three sites of: under the capsule of right kidney (K-site), the leg muscle (L-site,) and the back muscle (B-site) in the same animal. The grafted ovarian tissues were studied with both functional and histological techniques. Finally, body wall and skin incision were closed after suturing (Figure 1).

**Transplantations at different body sites**

In the same animal the kidney was exteriorized, after a dorso-horizental incision in the skin and body wall at the right side. A hemi-ovary was selected randomly and inserted under the right kidney capsule (K-site) through a tiny hole using watchmakers' forceps. For L-site transplantation, in the same animal a small incision was made in the skin on the quadriceps femoris muscle fascia of the right leg. Muscle of tight was exteriorized. A hole of 3-5mm deep was made in the L-site using the watchmakers' forceps and hemi-ovary was inserted into anterior muscles of thigh. In addition, in the same animal a small incision was made in the skin on back muscles (B-site). Muscle was exteriorized. A hole of 3-5 mm deep was made in the B-site, and hemi-ovary was inserted into the back muscles (Figure 1).

**Gonadotropin treatment in transplanted animals**

The end results of stimulated transplanted animals (experimental) were compared with unstimulated transplanted animals (control). Therefore, 1 week after grafting, ovarian stimulation was started by injection of 1IU FSH (rFSH; Merck) i.p., given every other day for 2 weeks. Finally, two doses of 5IU FSH was given i.p. followed by 1 dose of 5IU hCG i.p., 48h later. Animals were sacrificed by cervical dislocation 14h after hCG injection. Then, the grafts from K, B, and L sites were recovered for further analysis (5).

**In vitro maturation (IVM)**

All recovered grafts and control ovarian tissues were collected in α- minimal essential medium (α-MEM) supplemented with 10% fetal bovine serum (FBS, Gibco, UK). All grafted ovaries were dissected under a stereoscope using 29-gauge needle to retrieve all oocytes. Oocytes were denuded with 80 IU hyaluronidase (Sigma, USA) and mechanical pipetting. The immature oocytes were transferred to IVM medium (21). Immature oocytes were incubated in 20 µl droplets of α-MEM medium supplemented with 5% FBS and 100m IU/ml recombinant FSH (rFSH; Merck) at 37^o^C with 5% CO_2_ and 95% air with high humidity. Oocytes were observed under a stereomicroscope after 48h to determine maturity (Figure 1).

**Histology and follicle counting**

Histological assessment was carried out on grafted hemi-ovaries and non-grafted control. Two weeks after transplantation, the animals were killed by cervical neck dislocation. Ovarian grafts were removed from the kidney, back muscle, and quadriceps femoris muscle, as well as control group. The ovarian tissues were fixed in bouin's, then embedded in paraffin wax, serially sectioned at 5µm and stained with hematoxylin and eosin (H&E; Merck, Germany). 

The sections were examined serially for the present of follicles: (i) primordial follicles with one layer of flattened granulosa cells surrounding the oocyte; (ii) primary follicles with one layer of cuboidal granulosa cells; (iii) preantral follicles with two or more layers of granulosa cells with no antrum; (iv) antral follicles with an antral cavity. In order to avoid counting follicles more than once, only follicles were counted in the sections with visible oocyte nuclei. H&E staining was used to study the quality and grafts integration within the surrounding tissue (2, 4) (Figure 1).

**Statistical analysis**

Follicle counts and maturation rates are presented as mean±SD. The mean numbers of follicles were compared using a Kruskal-Wallis rank test and Mann-Whitney test. p<0.05 was considered statistically significant.

## Results

The overall total follicle count was significantly higher in K-graft (72.1%) than in B and L-grafts (27.9% and 0%), respectively. In particular, the percentage of primary follicles was significantly higher in K-graft (21.5%) compared to B and L-grafts (33.9% and 0%), respectively. Also, the data presented that the mean number of preantral follicles was significantly higher in K-graft compared to other grafts. In general, the total number of follicles in L-grafts was lower than B and K-grafts. The results also demonstrated that there was no antral follicle development in B- and L-grafts. Importantly, the follicular development to the antral stage was only observed in K-site model. 

There was significant differences in the number of preantral follicles between K, B, and L-grafts with control mice (p=0.032, p=0.016) (Table I). As presented in table II, the transplantation survival rate in the K-graft was more successful than the B- and L-graft groups (p=0.008, p=0.016). The number of retrieved immature oocytes in the K-grafts was significantly higher than other groups as well. In the present study, 41.7% of the oocytes were matured in control group after application of IVM. The oocyte maturation rate post IVM was 37.5% when ovarian tissues were transplanted to K site. This was higher when compared to L- and B-grafts (26.5% and 14.7%), respectively. Importantly, the highest rate of oocyte degeneration was noticed in B site (Table II).

**Table I:** Number of follicles in the hemi-ovaries to the back muscle (B-site), under kidney capsule (K-site), and leg muscle (L-site) in the same recipients

**Groups**	**Control **	**Kidney **	**Back **	**Leg**
primordial	0.6 ± 0.57 (4.7)	0 ± 0 (0)	0 ± 0 (0)	0 ± 0 (0)
primary	4.3 ± 1.5 (23.4)	0.6 ± 0.57 (21.5) a	0.3 ± 0.57 (33.9) a	0 ± 0 (0)
preantral	4.6 ± 4.6 (37.2)	0.6 ± 0.57 (21.5) a	0.6 ± 1.1 (66.1) a	0 ± 0 (0)
antral	6.3 ± 2.3 (34.7)	1.6 ± 1.5 (57) a	0 ± 0 (0)	0 ± 0 (0)

Number are presented as mean±SD (%).

p<0.05 is statistically significant (Kruskal-Wallis rank test)

a :Significant difference percentage with control group (p=0.032, p=0.016).

**Table II:** The rates of maturation oocytes in the hemi-ovaries to the back muscle (B-site), under kidney capsule (K-site), and leg muscle (L-site) in the same recipients in four groups in IVM

**Groups**	**Control (n=2)**	**Kidney (n=148)**	**Back (n=42)**	**Leg (n=8)**
MII	1.5±0.7 (41.7)	1.95±1.6 (37.5)	0.8±0.9 (14.7) a	0.7±0.5 (26.5) a
MI	0(0)	1.38±1.4 (15.3) a	0.1±0.3 (1.1)	0.5±0.5 (25) a
GV	0.5±0.7 (12.5)	2.95±2.3 (46.1) a	2.5±2.9 (43.3) a	0.5±0.5 (25) a
Degenerated	1.5±0.7 (45.8)	0.76±1.04 (1.1)	0.8±0.6 (40.9) a	0.25±0.5(23.5)

Number are presented as mean±SD (%).

p<0.05 is statistically significant (Chi-square test)

GV: germinal vesicle.

MI: Metaphase I.

MII: Metaphase II.

N: oocytes total number.

a : Significant difference percentage with control group (p=0.008, p=0.016).

**Figure 2:**
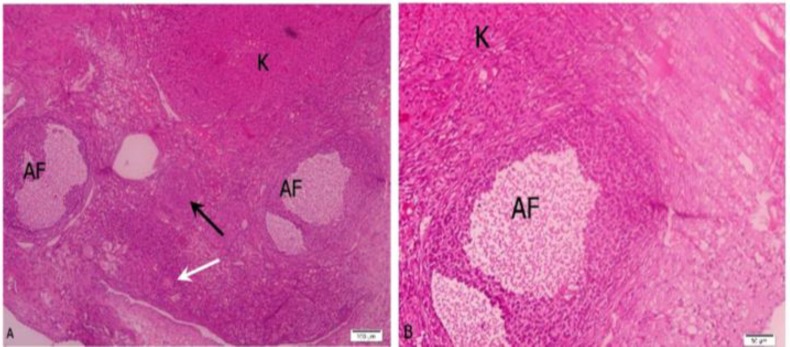
Mouse ovary grafted under the kidney capsule.

A: Note the presence of primary follicle (white arrow), preantral follicle (black arrow) and antral follicle (AF) in grafted ovarian tissue. K= kidney tissue. ) Bar=100µm(.

B: A section from kidney-graft Antral follicles (AF), kidney tissue (K). (Bar=50µm).

**Figure 3:**
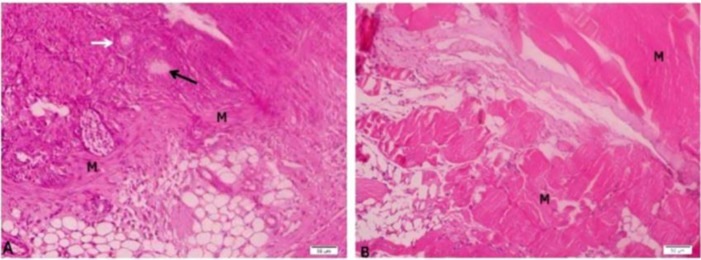
Mouse ovary grafted under the fascia of the quadriceps femoris and back muscles.

A: Back muscles-graft. Follicles in early stages were present. primary follicles (white arrow) and preantral follicles (black arrow). M= muscle. )Bare=50µm(

B: quadriceps femoris muscle-graft. No follicles development in L-graft. M= muscle. (Bar=50µm).

**Figure 4:**
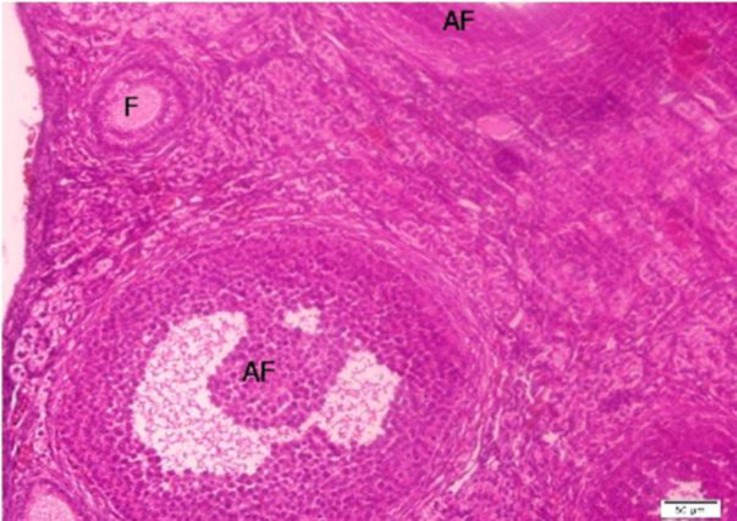
The normal ovarian cortex of control. primary follicle (F) and antral follicles (AF). Bar=50µm.

## Discussion

In this study, mice ovaries were transplanted into three different locations to determine the most suitable site for grafting. In addition, the follicular survival and oocyte maturation rates 2 weeks post transplantation were assessed. The histological survey confirmed that better follicular survival was observed in the K-site graft rather than in the B-site. These results are different to Abir *et al* who reported an improvement in post transplantation survival of human ovarian tissue into back muscle of mice (18). However, Dissen *et al* showed that kidney has a rich blood supply and high concentration of angiogenic growth factors, which makes it more suitable for grafting (19). In recent years, the kidney capsule has become popular as a feasible transplantation site, but difficult to access it for the recovery of oocytes from the grafts (6). Another study confirmed a better survival of follicles in the B-site than in the K-site grafts in mice (5). 

Our results are similar to those of Li *et al* who confirmed that follicular development was not satisfactory and the follicle density in the back muscle grafts was noticeably decreased in mice (20). Moreover, a number of follicles changed their normal morphology, and the majority of follicular oocytes were degenerated. Also, Kagawa *et al* grafted the porcine ovarian tissue under the capsule of kidney in mice. The findings revealed that follicles in grafted ovarian tissue grew rapidly and grafts had abundant capillary vessels (21). Also, Kaneko *et al* studied oocyte maturation and fertilizing ability in a model of ovarian xenografting. They transplanted ovarian tissue with many antral follicles were visible under kidney capsule (22). Our findings are consistent with previous study that reported increased follicular survival after grafting under the kidney capsule compared with grafting to subcutaneous sites in xenografted ovarian tissue (23). The influence of the oocyte quality from murine ovarian tissue grafted into bursal cavity, kidney capsule and subcutaneous was investigated. The results showed that a significantly more oocytes were recovered from the ovaries grafted under the kidney capsule (8). 

Furthermore, we confirmed better survival of follicles in the B- than in the L-site graft. Our results agree with Terazono *et al* who transplanted ovarian tissues under the fascia of the quadriceps femoris, thoracholumbar, and deltoid muscles. The histological examination revealed follicles at different stage of development, and a visible antral was observed under the thoracholumbar muscle (9). By contrast to our finding, Terazono *et al* studied the canine ovarian tissue autografted to quadriceps femoris muscle fascia, kidney capsule and gastrosplenic ligament (6). 

Their results showed that quadriceps femoris muscle fascia may be as suitable as the kidney capsules as a graft site. In our study, we could not confirm follicular development to the primordial stage. These results were consistent with those of Liu *et al* who transplanted newborn mouse ovaries under the kidney capsule (4). Their results indicated almost half of the total content of primordial follicle loss. In addition, Reynaud et al assumed that oocyte degeneration is the cause of the death within the primordial follicles (24). 

In addition, Dolmans *et al* studied short-term transplantation of isolated human ovarian in the right ovarian bursa of mice. They revealed a significant decrease in the proportion of primordial follicle compared with un-grafted tissue (25). The other goal of this study was to find out the transplantation success rate and IVM of oocytes in 3 different locations to determine the most suitable site for grafting. We found that when ovaries were transplanted to K-site, significant difference in oocyte maturation was seen when compared with other sites. Also, transplantation survival in K-site was satisfactory. According to Israely *et al* after ovarian graft into wound site, 71% of the oocytes were matured (26). 

In contrast with our study, previous report showed that 93% of MI oocytes become matured in B-sites graft. The mean maturation in our study was significantly lower to value attained by Solimani *et al* (5). Li *et al* showed that from 163 germinal vesicle oocytes retrieved from B-site, 146 oocytes (89%) reached to MII stage after IVM (20). It has been shown that ovarian transplantation is a successful option for patient with specific situation. But, this technology could not obtain significant mature oocytes after IVM in our study. It is not clearly established yet as which site yields the best results after ovarian tissue transplantation. However, we conclude that kidney capsule is superior to leg and back muscles for ovarian tissue grafting in mice. The grafts to the thigh and back muscles did not highly support the early follicle development and oocyte maturation in-vitro.

## Conflict of interest

There is no conflict of interest for authors in this research
